# Phosphoproteomic Analysis Reveals Differences in Intercellular Spread Among Feline Herpesvirus Type 1 Mutants

**DOI:** 10.3390/vetsci12121185

**Published:** 2025-12-11

**Authors:** Weiwei Lin, Xianglin Zhang, Qian Jiang, Hongtao Kang, Yijing Li, Honglin Jia, Jiasen Liu

**Affiliations:** 1 State Key Laboratory for Animal Disease Control and Prevention, Harbin Veterinary Research Institute, Chinese Academy of Agricultural Sciences, Harbin 150069, China (W.L; 17873772373@163.com (X.Z.); jiangqian@caas.cn (Q.J.); kanghongtao@caas.cn (H.K.); jiahonglin@caas.cn (H.J.); 2 College of Veterinary Medicine, Northeast Agricultural University, Harbin 150030, China

**Keywords:** FHV-1, phosphoproteomics, differential phosphorylation, GO, KEGG

## Abstract

Feline herpesvirus-1 (FHV-1) is taxonomically classified within the family Herpesviridae, subfamily Alphaherpesvirinae, genus Varicellovirus, and species Felid alphaherpesvirus 1. The genome of FHV-1 is 135,797 bp in length and encodes 74 proteins. Among these proteins, serine/threonine protein kinase (pK) and thymidine kinase (TK) have been identified as potential virulence factors in alphaherpesviruses, although these kinases are dispensable for viral replication. As kinases, regulating phosphorylation modification is one of their functions, while the mechanism by which phosphorylation modification affects cell physiological functions and thereby influences viral replication remains unclear. In this study, we generated pK- and TK-deficient FHV-1 mutants by CRISPR/Cas9-mediated homologous recombination. The pK-deficient virus produced significantly smaller plaques than the TK-deficient virus. The replication kinetics of the pK-deficient virus were attenuated in multistep growth compared to the TK-deficient virus. These results indicate that deletion of the pK gene markedly reduces the replicative capacity of FHV-1. We applied data-independent acquisition (DIA) quantitative proteomics to profile changes in global protein expression and phosphorylation in F81 cells upon infection with TK^−^, pK^−,^ and wild-type FHV-1 strain. The pK-deficient virus exhibited 3632 differentially phosphorylated proteins containing 11,936 modification sites; the TK-deficient virus showed 4529 differentially phosphorylated proteins with 19,225 phosphorylation sites. Functional characterization through Gene Ontology (GO) and Kyoto Encyclopedia of Genes and Genomes (KEGG) enrichment analyses identified significant involvement of phosphoproteins in spliceosome pathways in pK-deficient virus and ATP-dependent chromatin remodeling pathway in TK-deficient virus. Notably, several splicing regulators—including Ess-2 and CDK13, which modulate host spliceosomal function—displayed significantly reduced phosphorylation levels in pK-deficient viruses. A significant enrichment of ATP-dependent factors, such as SMARCA5 and RSF1, was observed in the TK-deficient virus. To our knowledge, this is the first investigation into the effects of FHV-1 infection on the host cell phosphoproteome. These data offer new insights into the phosphoregulatory circuits and signaling networks triggered by FHV-1 and may enhance our understanding of the FHV-1 replication mechanism.

## Simple Summary

This study aimed to elucidate how feline herpesvirus type 1 (FHV-1) serine/threonine protein kinase (pK) and thymidine kinase (TK) regulate viral replication through host protein phosphorylation. Using CRISPR/Cas9-generated pK^−^ and TK^−^ deletion mutants, we demonstrated that pK deletion resulted in significantly reduced plaque size and impaired replication capacity. Phosphoproteomic profiling revealed that pK deficiency specifically altered phosphorylation of spliceosome-associated proteins, whereas TK deletion predominantly affected the ATP-dependent chromatin remodeling pathway. Based on the experimental data, we hypothesize that PK and TK may regulate FHV-1 replication through distinct host signaling networks. Our work provides novel insights into FHV-1 pathogenesis and identifies potential targets for antiviral development.

## 1. Introduction

Feline herpesvirus type 1 (FHV-1) is the primary causative agent of feline viral rhinotracheitis (FVR), a disease mainly characterized by upper respiratory and ocular signs [1]. FHV-1 belongs to the genus Varicellovirus, subfamily Alphaherpesvirinae, family Herpesviridae [2,3]. It is an enveloped virus with a linear double-stranded DNA genome organized into unique long (UL) and unique short (US) regions, flanked by internal and terminal repeats (IRS, TRS), forming the structure UL-IRS-US-TRS [4,5]. The complete genome spans 135,797 bp and encodes 74 viral proteins, including 11 glycoproteins [6].

Notably, the US3 gene encodes a serine/threonine protein kinase (pK) involved in virion assembly, cytoskeletal remodeling, and evasion of host innate immunity [7]. The pK gene is essential for efficient viral replication in vitro, as its deletion impairs the growth of multiple herpesviruses, including FHV-1 [8]. Deletion of US3 attenuates FHV-1 virulence, enhances interferon production, impairs establishment of latency in the trigeminal ganglion, and reduces subsequent viral shedding [9]. In addition to the US3 protein, thymidine kinase (TK) has been implicated in herpesvirus pathogenicity. While TK is not required for in vitro replication or immunogenicity in FHV-1, it is critical for replication and dissemination of pseudorabies virus in the central nervous system [10]. Partial or complete deletion of the TK gene has been shown to reduce virulence and neuroinvasiveness in several herpesviruses [11].

Protein phosphorylation represents a fundamental post-translational modification that orchestrates crucial biological processes, including signal transduction, molecular interactions, and enzymatic activity regulation [12,13]. This covalent modification significantly expands protein functional diversity, with approximately 30% of the proteome undergoing phosphorylation at one or more residues [14]. These modifications are catalyzed by protein kinases, which constitute nearly 2% of the eukaryotic genome [15]. In mammalian systems, phosphorylation primarily occurs on serine, threonine, and tyrosine residues. The reversible nature of this process—tightly regulated by kinases and phosphatases—enables dynamic control of cellular signaling networks. Substantial evidence demonstrates the indispensable role of phosphorylation in diverse cellular processes, including inflammasome assembly and activation [16], apoptosis [17], oncogenic proliferation [18,19], and virus–host interactions [20].

Previous studies have shown that in alphaherpesviruses, such as pseudorabies virus (PRV) and herpes simplex virus 1 (HSV-1), the pK protein directly phosphorylates core components of the m^6^A mRNA methyltransferase complex, thereby suppressing its methylation activity [21]. In Marek’s disease virus, the pK kinase controls replication and disease progression via direct phosphorylation of host HDAC1 and HDAC2 [22]. TK catalyzes the monophosphorylation of nucleoside analogues. Currently, the phosphorylation-related functions of the pK and TK genes remain incompletely characterized. Moreover, global phosphoproteomic changes induced by FHV-1 infection remain uncharacterized.

In this study, we generated targeted pK^−^ and TK^−^ mutants of FHV-1 using CRISPR/Cas9-mediated homologous recombination. Our assessment of viral growth curves and plaque sizes yielded results consistent with these earlier reports. These observations prompted us to further investigate the mechanisms underlying the differential replication efficiency among the mutant strains. Then, we performed phosphoproteomic sequencing and employed liquid chromatography-tandem mass spectrometry (LC-MS/MS) to profile host protein phosphorylation in response to infection with wild-type FHV-1 and its gene-deletion mutants. All experiments were conducted in three independent replicates to ensure reproducibility. Using the wild-type infection as a reference, we identified differentially phosphorylated proteins in mutant strains and performed functional annotation through Gene Ontology (GO), Kyoto Encyclopedia of Genes and Genomes (KEGG) pathway, and subcellular localization analyses. Our findings may elucidate the FHV-1 infection process and the mechanisms by which host phosphorylation dynamics block and limit viral replication.

## 2. Materials and Methods

### 2.1. Cells and Virus

Feline kidney cells (F81) were maintained in Dulbecco’s Modified Eagle Medium (DMEM) supplemented with 10% (*v/vv/v*) fetal bovine serum (FBS), 100 U mL^−1^ penicillin, and 100 μg mL^−1^ streptomycin, and cultured at 37 °C in a humidified atmosphere containing 5% CO_2_. Feline herpesvirus-1 strain QD-1 was isolated in this laboratory from the nasal swab of a kitten exhibiting clinically confirmed feline viral rhinotracheitis. For viral propagation, confluent F81 monolayers were inoculated with FHV-1 at a multiplicity of infection (MOI) of 0.01. When approximately 90% cytopathic effect (CPE) was observed, the culture supernatants were harvested and clarified for subsequent titration.

### 2.2. Selection of sgRNAs

Single-guide RNAs (sgRNAs) targeting the TK and pK sequences were designed using the online CRISPR design tool (https://www.zlab.bio/, Feng Zhang’s Lab). The sgRNAs are listed in Table 1. The PX330 plasmid was digested using BbsI, and the CRISPR/Cas9-sgRNA plasmids were constructed. All of the constructs in this study were verified by sequencing.

### 2.3. Construction of Recombinant Transfer Vectors

For deletion of the pK gene of FHV QD-1 by homologous recombination, a transfer vector was constructed by using two segments flanking the pK gene, pK-L and pK-R, as the recombination homologous arms. The left and right homology arms (pK-L and pK-R) were PCR-amplified from FHV QD-1 genomic DNA using the primer sets pKL-F/pKL-R and pKR-F/pKR-R, respectively. The EGFP expression cassette under the control of the CMV promoter and SV40 poly(A) signal was amplified from pEGFP-N1 with primers pK-EGFP-F and pK-EGFP-F using the primer set pK-EGFP-F and pK-EGFP-R. The pK-L and pK-R homology arms together with the EGFP expression cassette were directionally inserted between the BamHI and EcoRI sites of pUC19 to generate the final transfer vector pUC19-pKLR-EGFP. Following the identical cloning strategy, pUC19-TKLR-EGFP, a transfer plasmid designed for TK gene deletion, was constructed by inserting the corresponding TK-L and TK-R homology arms together with the EGFP expression cassette between the BamHI and EcoRI sites of pUC19. Primers used are listed in Table 2.

### 2.4. CRISPR/Cas9-Mediated Gene Deletion and Recombinant Virus Purification

F81 cells were transfected with 2 μg px330-pK-sgRNA and pUC19-pKLR-EGFP using PEI reagent according to the manufacturer’s instructions. After 24 h, cells were inoculated with the FHV-1 QD-1 strain at an MOI of 0.01. At 48 h post-infection, cells and supernatant were collected and lysed by three consecutive freeze–thaw cycles to liberate intracellular virions. Recombinant FHV-1 was purified from the cell lysates by plaque purification in F81 cells overlaid with 1% low-melting-point agarose and 2% FBS. Following three rounds of plaque purification, EGFP-expressing plaques were readily visualized by fluorescence microscopy. The DNA of recombinant viruses was extracted using a TIANamp Genomic DNA Kit (TIANGEN, Beijing, China) and amplified using the specific primers pK-F1/pK-R1. The PCR product was sequenced, and the pK gene deletion and EGFP insertion were confirmed. The clonally purified, EGFP-expressing recombinant virus was named FHV QD-1 pK^−^/EGFP^+^. Similarly, the pUC19-TKLR-EGFP and px330-TK-sgRNA were transfected into F81cells, and the cells were infected with FHV-1 QD-1 strain 24 h after transfection. FHV QD-1 TK^−^/EGFP^+^ was screened by three rounds of purification and identification via PCR using primer pairs TK-F1/TK-R1 and sequencing. Primers used are listed in Table 3.

### 2.5. Growth Kinetics and Plaque Assays of the Recombinant FHV-1

F81 cells were cultured in six-well plates and inoculated with FHV QD-1, pK^−^/EGFP^+,^ and TK^−^/EGFP^+^ at 0.01 MOI, respectively. The supernatants and cells were collected at the indicated intervals. The virus titers were determined using the Reed–Muench method. Confluent F81 monolayers in six-well plates were inoculated with FHV QD-1, pK^−^/EGFP^+^, or TK^−^/EGFP+ at 37 °C for 2 h, then overlaid with DMEM supplemented with 1% (*w*/*v*) low-melting-point agarose and 2% (*v/vv/v*) FBS, and incubated at 37 °C under 5% CO_2_ for 72 h. The six-well culture plates were stained with 1% crystal violet in methanol for 1 h at room temperature before the plaques were observed.

### 2.6. Samples for LC-MS-MS Analysis

F81 cells were seeded in 75 cm^2^ cell culture flasks and cultivated until achieving 80% confluency. The growth medium was removed, and cells were washed three times with sterile phosphate-buffered saline. Cells were infected with FHV QD-1, pK^−^/EGFP^+^, and TK^−^/EGFP^+^ at 0.01 MOI, incubated at 37 °C with 5% CO2, and harvested after 12 h for LC-MS-MS analysis. Uninfected cells were incubated in DMEM alone as a non-infected control (NC) group. To ensure adequate coverage of phosphosites, three biological replicates (a total of nine samples), including three FHV QD-1, three pK^−^/EGFP^+^, and three TK^−^/EGFP^+^, were used.

### 2.7. Protein Extraction

Cells were resuspended in lysis buffer (8M urea, 1% protease inhibitor, 1% phosphatase inhibitor, 3 μM Trichostatin A, and 50 mM Nicotinamide) and sonicated three times on ice using a high-intensity ultrasonicator, and precipitates were removed by centrifugation at 12,000× *g* for 10 min at 4 °C. The supernatant was collected following the manufacturer’s protocol, and protein concentrations were quantified using a bicinchoninic acid (BCA) assay.

### 2.8. Protein Digestion and Phosphopeptide Enrichment

Following quantification, protein aliquots were subjected to reduction with 10 mM dithiothreitol (DTT) at 37 °C for 1 h and alkylation with 50 mM iodoacetamide (IAA) in the dark at room temperature for 1 h. Excess IAA was quenched by the addition of 5 mM DTT with incubation at room temperature for 10 min. The samples were then transferred to 30-kDa molecular weight cut-off (MWCO) filters and centrifuged. The filter units were subsequently washed twice with 100 μL of UA buffer (8 M urea in 100 mM Tris-HCl, pH 8.5) and three times with 100 μL of 50 mM ammonium bicarbonate (NH_4_HCO_3_) to remove residual denaturant. After buffer exchange, trypsin was added at a 1:50 (*w*/*w*) enzyme-to-protein ratio in 50 mM NH_4_HCO_3_, and digestion proceeded at 37 °C for 16–20 h. The resulting peptides were desalted using C18 cartridges, lyophilized, and reconstituted in 40 μL of 0.1% formic acid. Peptide concentration was determined by absorbance at 280 nm. For phosphopeptide enrichment, the HiSelect^TM^TiO_2_ Phosphopeptide Enrichment Kit (Thermo Fisher Scientific, Waltham, MA USA, A32993) was used according to the manufacturer’s protocol. The enriched phosphopeptides were lyophilized and stored in 0.1% formic acid for subsequent LC-MS/MS analysis.

### 2.9. Nanoflow Ultra-High Performance Liquid Chromatography (UHPLC)

Chromatographic separation was performed using a nanoflow ultra-high performance liquid chromatography (UHPLC) system. The mobile phases consisted of 0.1% formic acid in water (phase A) and 0.1% formic acid in 80% acetonitrile (phase B). After equilibrating the column with 100% phase A, samples were loaded via an autosampler onto a trapping column and subsequently separated on an analytical column. The mass spectrometry gradient program is detailed in Table 4.

### 2.10. Mass Spectrometric Analysis

Following separation by nanoflow liquid chromatography, samples were analyzed using an Orbitrap Astral mass spectrometer (Thermo Scientific) in positive ion mode. The total analysis time was 24 min. Detailed mass spectrometry parameters are listed in Table 5.

### 2.11. Mass Spectrometry Data Processing

A spectral library was constructed by searching against a protein sequence database in the FASTA format, which included the Felis catus (host) and FHV-1 (virus) proteomes. Subsequent analysis of data-independent acquisition (DIA) data was performed using Spectronaut Pulsar software 20 (Biognosys AG, Schlieren, Switzerland). Following database searching against the feline-specific UniProt database, raw LC-MS/MS files were imported into Spectronaut for spectral library generation and comprehensive data processing. In order to test the reproducibility of experiments, phosphoprotein ratios in each replicate were quantified based on the summed intensity of the matched spectra. Pearson correlation coefficient and average relative standard deviation (RSD) were explored to evaluate the reproducibility of protein relative quantitation.

### 2.12. Data Filtering and Preprocessing

To ensure data quality, proteins and peptides were retained only if they were detected in more than 50% of samples within any experimental group. For the filtered dataset, missing values were imputed using the mean of respective sample groups. The resulting data then underwent median normalization followed by log_2_ transformation to generate a finalized set of high-confidence phosphoproteins and phosphopeptides.

### 2.13. Differential Expression Analysis

For the identification of significantly differentially phosphorylated peptides, we applied the following criteria: a phosphopeptide was considered significantly upregulated if it exhibited a fold change (FC) > 1.2 with a *p*-value < 0.05 (Student’s *t*-test), and downregulated if it exhibited a fold change (FC) < 0.5 with a *p*-value < 0.05. The numbers of up- and downregulated phosphopeptides for each comparison were determined based on these thresholds.

### 2.14. Bioinformatic Analysis and Visualization

Phosphorylation motif enrichment was analyzed using the MOMO tool from the MEME Suite. Functional characterization of the phosphoproteome was performed through integrated bioinformatic approaches, including Gene Ontology (GO) term enrichment, Kyoto Encyclopedia of Genes and Genomes (KEGG) pathway analysis, and the OmicShare Tools platform. Data visualization was implemented using multiple specialized tools: density plots were generated with the ggplot2 package in R, protein correlation patterns were visualized using the corrplot package (v0.84) in R, and bar charts, along with pie charts, were created using GraphPad Prism (v8.0.2). The phosphorylated protein analysis was performed in three independent experiments. Data are presented as the mean ± standard deviation unless otherwise specified. Statistical significance was defined as *p* < 0.05 (GraphPad 6/ SPSS 20).

## 3. Results

### 3.1. Construction and Identification of the FHV-1 Mutant via CRISPR/Cas9-Mediated Homologous Recombination

**Figure 1:**
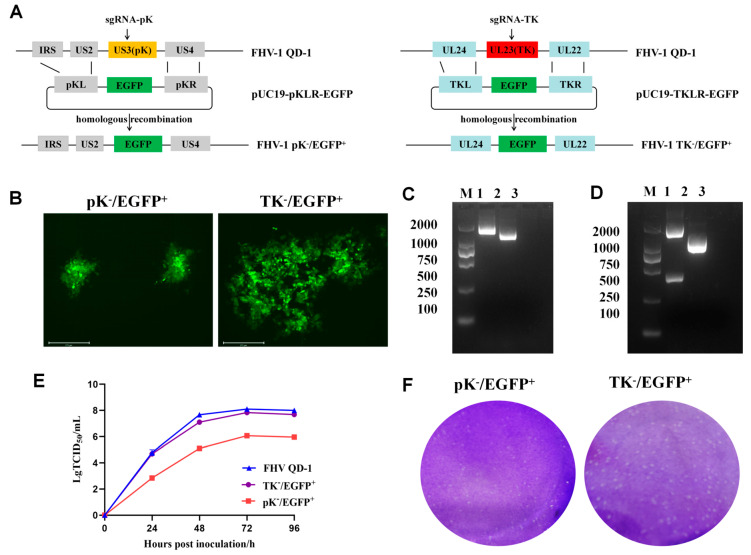
Construction and identification of recombinant FHV-1. (**A**) Schematic diagram of the recombinant FHV-1 development strategy. (**B**) Plaque purification of pK^−^/EGFP^+^ and TK^−^/EGFP^+^ in agarose-DMEM plates. (**C**) PCR confirmation of the pK gene deletion and the EGFP insertion. Lane M: DNA Molecular Weight Standard (DL2000); Lane 1: pK^−^/EGFP^+^; Lane 2: FHV-1 WT QD-1; Lane 3: blank control. (**D**) PCR confirmation of the TK gene deletion and the EGFP insertion. Lane M: DNA Molecular Weight Standard (DL2000); Lane 1: TK^−^/EGFP^+^; Lane 2: FHV-1 WT QD-1; Lane 3: blank control. (**E**) Multiple-step growth curves of FHV QD-1, pK^−^/EGFP^+^, and TK^−^/EGFP^+^ in F81 (0.01 MOI). (**F**) Plaque morphologies and sizes of F81 cells inoculated with the pK^−^/EGFP^+^, and TK^−^/EGFP^+^.: Original PCR/Gel Electrophoresis pictures see Figures S1 and S2.

Utilizing CRISPR/Cas9-mediated homologous recombination (Figure 1A), we generated a series of FHV-1 mutants. F81 cells were co-transfected with the pKL-CMV-EGFP-bGH polyA-pKR fragment and a sgRNA specific for the pK gene. At 24 h post-transfection, the cells were infected with the FHV-1 QD-1 strain at an MOI of 0.01. Following the emergence of green fluorescent plaques exhibiting characteristic herpesvirus cytopathic effects, the pK^−^/EGFP^+^ virus was isolated through three rounds of plaque purification (Figure 1B). The identity of the recombinant virus was then verified by PCR amplification and sequencing, which confirmed a distinct amplification profile compared to the wild-type strain. The size of the pK^−^/EGFP^+^ target gene is 1700 bp, while that of FHV-1 is 1200 bp. (Figure 1C). Finally, the sequence-confirmed recombinant virus, in which EGFP replaces the pK gene, was harvested and stored for subsequent experiments. The TK^−^ mutant was constructed following an identical procedure. The PCR identification results indicate the size of the pK^−^/EGFP^+^ target gene is 1700 bp, while that of FHV-1 is 1200 bp, which meets our expectations.

To assess the impact of pK or TK deletion on FHV-1 biological properties, we examined viral replication kinetics and cell-to-cell spread. Using the FHV-1 QD-1 strain as a control, multistep growth curve analysis revealed that the pK^−^/EGFP^+^ mutant replicated more slowly than both the wild-type QD-1 and TK^−^/EGFP^+^ strains, requiring an extended time to achieve CPE in F81 cells, the peak titer of the pK^−^/EGFP^+^ mutant reached 10^6^ TCID_50_/mL, significantly lower than the 10^8^.^3^ TCID_50_/mL attained by the wild-type FHV-1 QD-1 strain (Figure 1E). The data confirm that pK gene deletion reduces FHV-1 replicative fitness. As shown in Figure 1B, the pK^−^/EGFP^+^ mutant formed significantly smaller plaques than TK^−^/EGFP^+^, indicating that pK deletion impairs intercellular viral spread.

### 3.2. Global Detection of Phosphosites in F81 Cells Infected with FHV-1 Mutants

**Figure 2:**
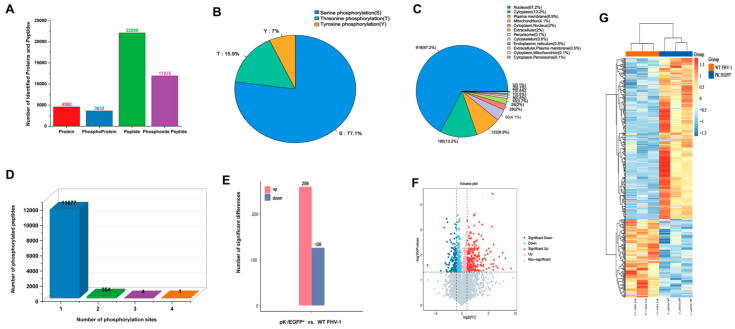
A quantitative overview of the phosphoproteomic analysis of phosphoproteins with differentially regulated phosphosites in pK^−^/EGFP^+^ and WT FHV-1 groups. (**A**) The number of phosphopeptides, phosphorylation sites, and phosphoproteins identified. (**B**) Quantitative statistics of the phosphorylation of serine, threonine, and tyrosine. (**C**) Prediction of the subcellular localization of phosphorylated proteins. (**D**) Statistical analysis of the number of modification sites on the phosphorylated peptide fragments. (**E**) Overview of differential phosphorylated phosphopeptides. (**F**) Volcano map analysis of the differential phosphorylated phosphopeptides. (**G**) Hierarchical clustering analysis of the differential phosphorylated phosphopeptides.

Analysis of phosphorylation modifications revealed both single-site and multisite events. To quantify these modifications, a comparative analysis between the pK^−^/EGFP^+^ and WT FHV-1 groups was performed. As illustrated in Figure 2A, this analysis identified 22,099 phosphorylated peptides, 11,935 phosphorylation sites, and 3632 phosphorylated proteins. Regarding the distribution of phosphorylation types, serine residues accounted for the majority (77.1%) of all identified sites, followed by threonine (15.9%) and tyrosine (7.0%) (Figure 2B). Subsequently, subcellular localization prediction indicated that these phosphorylated proteins were primarily distributed in the nucleus and cytoplasm (Figure 2C). Furthermore, analysis of the phosphorylated peptides revealed that 11,677 contained a single phosphorylation site, while 254, 4, and 1 peptides contained two, three, and four sites, respectively (Figure 2D).

**Figure 3:**
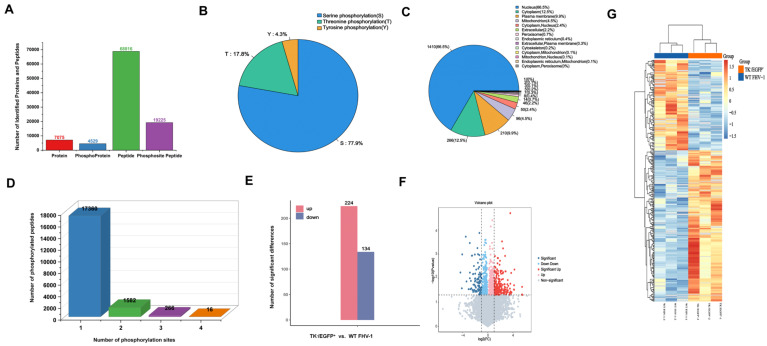
A quantitative overview of the phosphoproteomic analysis of phosphoproteins with differentially regulated phosphosites in TK^−^/EGFP^+^ and WT FHV-1. (**A**) The number of phosphopeptides, phosphorylation sites, and phosphoproteins identified. (**B**) Quantitative statistics of the phosphorylation of serine, threonine, and tyrosine. (**C**) Prediction of the subcellular localization of phosphorylated proteins. (**D**) Statistical analysis of the number of modification sites on the phosphorylated peptide fragments. (**E**) Overview of differential phosphorylated phosphopeptides. (**F**) Volcano map analysis of the differential phosphorylated phosphopeptides. (**G**) Hierarchical clustering analysis of the differential phosphorylated phosphopeptides.

In a parallel comparative phosphoproteomic analysis of the TK^−^/EGFP^+^ and WT FHV-1 groups, a larger dataset was obtained, comprising 68,816 phosphopeptides, 19,225 phosphosites, and 4529 phosphorylated proteins (Figure 3A). Consistent with the trend observed above, phosphoserine (pS) residues were again predominant (77.9%), with phosphothreonine (pT) and phosphotyrosine (pY) constituting 17.8% and 4.3% of the sites, respectively (Figure 3B). Regarding cellular distribution, the phosphoproteins in this dataset were predicted to localize primarily to the nucleus, cytoplasm, and plasma membrane (Figure 3C). Finally, the distribution analysis of phosphosites showed that 17,360 peptides featured a single site, whereas 1582, 266, and 16 peptides contained two, three, and four sites, respectively (Figure 3D).

To further explore the differential phosphorylation of host proteins in response to infection by the wild-type strain compared to the gene deletion strains. Following pK^−^/EGFP^+^ infection, 385 phosphorylated peptides were identified as significantly differentially expressed. Of these, 259 were significantly upregulated (fold change ≥ 1.5, *p* ≤ 0.05), while 126 were significantly downregulated (ratio ≤ 0.67, *p* ≤ 0.05) (Figure 2E). 224 upregulated and 134 downregulated differentially expressed proteins after TK^−^/EGFP^+^ infection (Figure 3E). The overall distribution of these significant changes is displayed in volcano plots (Figure 2F and Figure 3F). Subsequently, clustering analysis confirmed a clear separation between the phosphopeptides of the WT and gene deletion strain groups, underscoring the distinct impact of the viral genotype on the host phosphoproteome. Furthermore, among the differentially phosphorylated peptides, upregulated species were substantially more abundant than downregulated ones, a finding consistent with the trends observed in the volcano plots (Figure 2G and Figure 3G).

### 3.3. Analysis of Phosphorylated Peptide Motifs

**Figure 4:**
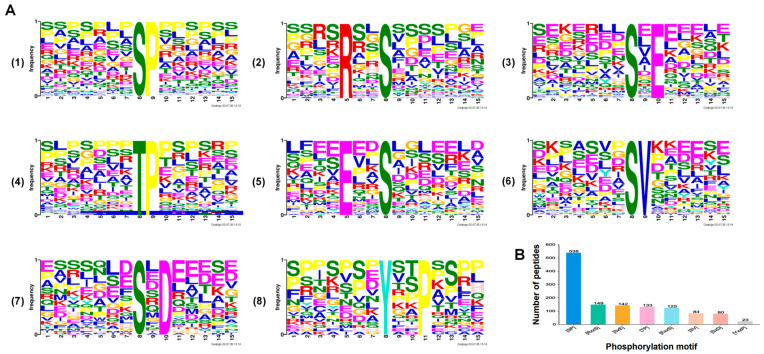
The phosphorylation motifs identified by the pK^−^/EGFP^+^ and WT FHV-1 groups. (**A**) Significantly enriched phosphorylation sequences. (**B**) The number of recognized peptides containing phosphorylated motifs.

**Figure 5:**
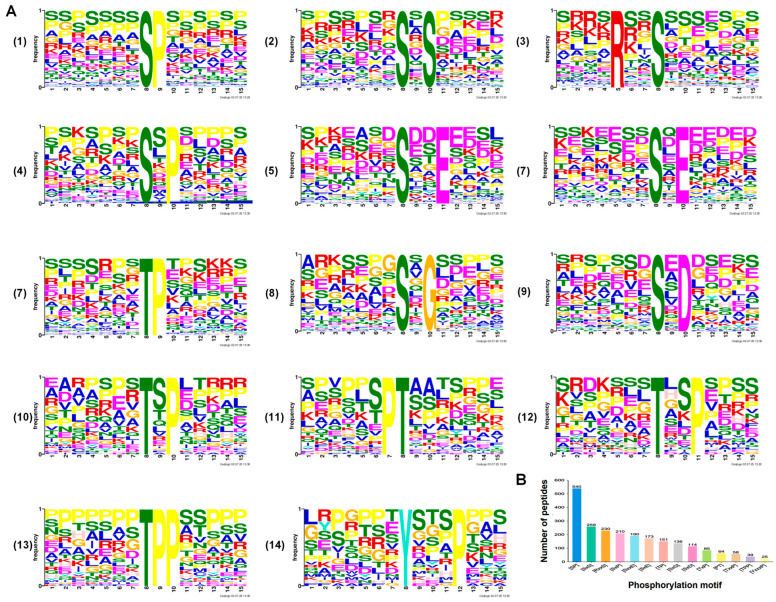
The phosphorylation motifs identified by the TK^−^/EGFP^+^ and WT FHV-1 groups. (**A**) Significantly enriched phosphorylation sequences. (**B**) The number of recognized peptides containing phosphorylated motifs.

To investigate the sequence conservation surrounding the phosphorylation sites, we analyzed the flanking regions spanning six amino acids upstream to six amino acids downstream of each modified residue. This analysis revealed conserved sequence motifs and aided in predicting potential cognate kinases. Specifically, motif analysis of the 1276 phosphopeptides identified in both the pK^−^/EGFP^+^ and WT FHV-1 groups showed that serine was the predominant phosphorylated residue (Figure 4A(1–3)(5–7)). Among these, six serine phosphorylation motifs were significantly enriched, with SP and RxxS being the most prevalent (Figure 4B). Furthermore, examination of all 2270 phosphopeptides identified 14 conserved motifs, including 9 phosphoserine (P-Ser) (Figure 5A(1–6)(8–9)) and 5 phosphothreonine (P-Thr) motifs (Figure 5A(7)(10–13)), in the TK^−^/EGFP^+^ and WT FHV-1 groups. These motifs exhibited distinct abundance patterns, with SP being the most widely distributed phosphoserine motif, followed by SxS and RxxS (Figure 5B).

### 3.4. Analysis of Phosphorylated Peptide Domain

**Figure 6:**
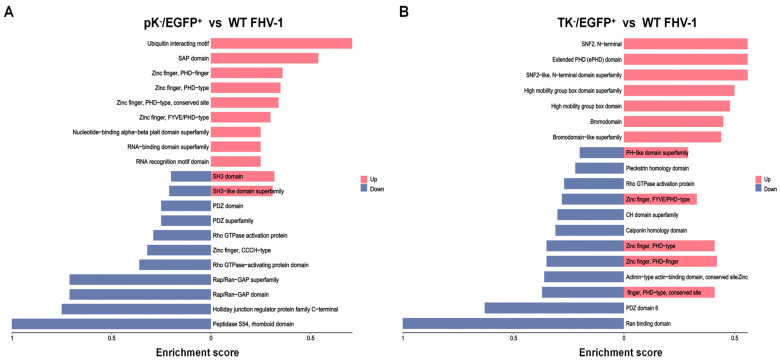
The structural domain prediction histograms. (**A**) The structural domain prediction histograms in the pK^−^/EGFP^+^ and WT FHV-1 groups. (**B**) The structural domain prediction histograms in the TK^−^/EGFP^+^ and WT FHV-1 groups.

In addition, domain prediction was performed on proteins harboring differentially expressed modified peptides. The phosphorylation levels of proteins associated with the RNA-binding domain superfamily and the RNA recognition motif (RRM) domain—both directly involved in RNA binding and processing—were observed to be elevated in the pK^−^/EGFP^+^ strain (Figure 6A). Upon phosphorylation, certain host RNA-binding proteins (RBPs) exhibit enhanced RNA-binding affinity or stability, enabling more efficient recognition and binding of viral RNA [23], followed by recruitment of degradation machinery for viral RNA clearance. Domain superfamilies implicated in chromatin remodeling and epigenetic regulation—including the SNF2 N-terminal, SNF2-like N-terminal, Bromodomain, and High Mobility Group box—were significantly upregulated in the TK^−^/EGFP^+^ strain (Figure 6B). Furthermore, subunits of many chromatin remodeling complexes, notably SWI/SNF, are regulated by phosphorylation. Phosphorylation upregulation directly enhances its ATPase activity or alters nucleosome positioning, thereby establishing an open chromatin environment at viral genome promoters to promote viral gene transcription [24,25]. Notably, the zinc finger PHD-type domain displayed distinct expression patterns between the mutants: it was upregulated in the pK^−^/EGFP^+^ strain, whereas in the TK^−^/EGFP^+^ strain, the extent of upregulation marginally surpassed that of downregulation. Given that the PHD zinc finger is a critical epigenetic reader of the histone code and plays essential roles in transcriptional regulation and DNA damage repair [26], its differential expression across viral strains suggests altered strategies in virus–host chromatin interactions during infection.

### 3.5. Functional Enrichment of Proteins with Differentially Regulated Phosphosites

**Figure 7:**
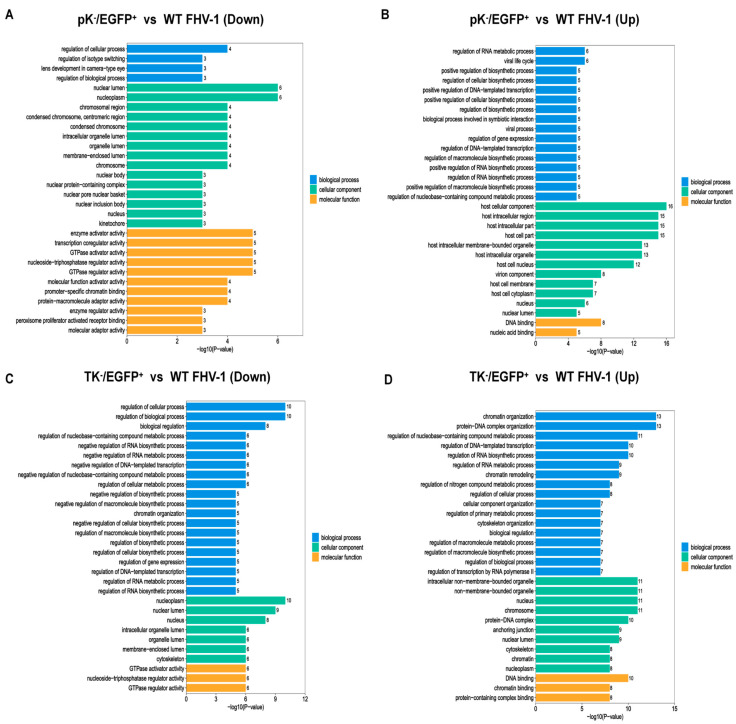
Gene Ontology functional enrichment analyses of proteins with differentially regulated phosphosites. (**A**) GO-based enrichment analysis of proteins with upregulated phosphosites in the pK^−^/EGFP^+^ and WT FHV-1 groups. (**B**) GO-based enrichment analysis of proteins with downregulated phosphosites in the pK^−^/EGFP^+^ and WT FHV-1 groups. (**C**) GO-based enrichment analysis of proteins with upregulated phosphosites in the TK^−^/EGFP^+^ and WT FHV-1 groups. (**D**) GO-based enrichment analysis of proteins with downregulated phosphosites in the TK^−^/EGFP^+^ and WT FHV-1 groups.

We performed Gene Ontology (GO) enrichment analysis on proteins associated with differential phosphorylation sites to systematically characterize their functional implications. The GO framework categorizes protein functions into three primary domains: Biological Process, Cellular Component, and Molecular Function. In the comparison between the pK^−^/EGFP^+^ and WT FHV-1 groups, proteins with downregulated phosphosites revealed a distinct functional profile. Specifically, within the Molecular Function category, the most significantly enriched terms included enzyme activator activity, transcription coregulator activity, GTPase activator activity, nucleoside-triphosphatase regulator activity, and GTPase regulator activity. Regarding cellular localization, the nuclear lumen and nucleoplasm were the most enriched Cellular Component terms. In the Biological Process domain, the top four enriched subcategories were regulation of cellular process, regulation of isotype switching, lens development in camera-type eye, and regulation of biological process (Figure 7A). Unlike the downregulated phosphosites, the enrichment results for proteins with upregulated phosphosites yielded distinct functional characteristics in both the Molecular Function and Cellular Component categories. Notably, DNA binding emerged as the most significantly enriched Molecular Function term, followed by nucleic acid binding. Within the Cellular Component category, the top five enriched terms were host cellular component, host intracellular region, host intracellular part, host cell part, and host intracellular membrane-bounded organelle (Figure 7B).

We next extended this analysis to the TK^−^/EGFP^+^ and WT FHV-1 comparison. For phosphoproteins with downregulated sites, GO enrichment again showed category-specific patterns. Nucleoplasm represented the most significantly enriched Cellular Component, with nuclear lumen ranking second. In parallel, regulation of cellular process and regulation of biological process were the primary terms enriched in the Biological Process category (Figure 7C). Conversely, proteins with upregulated phosphosites in this comparison displayed a contrasting functional signature. Specifically, the top three enriched Biological Process terms were chromatin organization, protein-DNA complex organization, and regulation of nucleobase-containing compound metabolic process. Meanwhile, the top four enriched Cellular Component categories were intracellular non-membrane-bounded organelle, non-membrane-bounded organelle, nucleus, and chromosome (Figure 7D).

**Figure 8:**
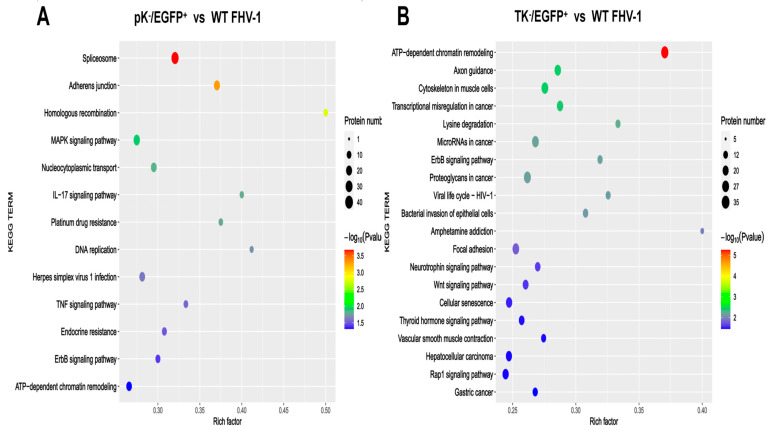
Kyoto Encyclopedia of Genes and Genomes (KEGG) pathway analysis of the differentially expressed proteins. (**A**) KEGG enrichment analysis of differentially expressed proteins in the comparison between the pK^−^/EGFP^+^ and WT FHV-1 groups (**B**) KEGG enrichment analysis of differentially expressed proteins in the comparison between the TK^−^/EGFP^+^ and WT FHV-1 groups.

To further explore the roles of differentially phosphorylated proteins and to uncover the molecular mechanisms underlying FHV-1 intercellular transmission, we used the Kyoto Encyclopedia of Genes and Genomes (KEGG) for pathway enrichment analysis. This approach identified distinct signaling and regulatory pathways across the mutant strains. A comparative analysis of viral mutants revealed distinct mechanisms of host manipulation. While the pK^−^/EGFP^+^ strain exhibited significant enrichment of the spliceosome pathway, implicating severe disruption of post-transcriptional regulation (Figure 8A), the TK^−^/EGFP^+^ mutant induced a major reprogramming of the host epigenetic landscape. This was evidenced by significant enrichment of the ATP-dependent chromatin remodeling pathway (Figure 8B), a feature markedly less pronounced in the pK^−^/EGFP^+^ strain.

## 4. Discussion

FHV-1 mutants with deletions in the pK or TK genes have been previously constructed and characterized in vitro [8,27]. In the present study, we also generated pK^−^ and TK^−^ deletion mutants and systematically characterized their biological properties. Our results recapitulated the established phenotype of the pK^−^ and TK^−^ mutant; the pK^−^ mutant produced smaller plaques and exhibited slower replication kinetics than the TK^−^ mutant. These are likely linked to its function in mediating nucleocapsid nuclear egress. Therefore, pK plays a critical role in promoting viral replication.

Viral replication emerges from complex host–pathogen interactions, with phosphorylation events orchestrated by protein kinases comprising a major regulatory axis in this process. The mechanistic basis by which the protein kinases pK and TK regulate phosphorylation during FHV-1 infection remains unclear. In this study, we systematically analyzed differentially regulated protein phosphorylation events following infection with distinct FHV-1 mutants to construct a comprehensive phosphoproteomic landscape. Our analysis identified 126 downregulated phosphorylation sites in the pK^−^ mutant and 134 in the TK^−^ mutant. Next, we performed a functional enrichment analysis on proteins with differentially regulated phosphorylation.

The pK^−^ mutant profoundly reprograms the host cellular environment, primarily by disrupting central gene regulatory machinery and activating immune-inflammatory pathways. Among these, the most pronounced alteration was observed in the spliceosome pathway. Consistent with this, we observed a concomitant shift in the phosphorylation status of key regulators of host gene expression and inflammation, such as Ess-2, CDK13, and STAT5A.

Ess-2 is an RNA-binding protein primarily involved in pre-mRNA splicing, a crucial step in the regulation of gene expression [28]. Notably, recent studies have shown that many viral infections lead to the reprogramming of host cell splicing, which can be attributed to direct viral manipulation, virus-induced immune responses, or cellular damage [29,30,31]. A well-established example of this viral strategy is the HSV-1-encoded protein ICP27, which interacts with SAP145, a critical component of the U2 snRNP complex within the spliceosome [32]. This interaction promotes the nuclear export of viral and select host transcripts, thereby facilitating viral replication and contributing to host shutoff. In line with these documented virus–spliceosome interactions, our findings are consistent with the notion that the host splicing machinery is involved in FHV-1 infection. Nevertheless, a direct functional characterization of Ess-2 in FHV-1 replication and host interaction remains essential. Beyond Ess-2, another key regulator of splicing is the kinase CDK13. CDK13 controls cell proliferation and differentiation by regulating key steps in gene expression [33]. Specifically, the RS motif in its N-terminal domain mediates interactions with spliceosomal components [34]. Furthermore, CDK13 controls splicing efficiency and fidelity by phosphorylating key splicing factors, thereby modulating their activity, localization, and interactions. However, it remains unknown whether CDK13 phosphorylation is functionally relevant to FHV-1 infection.

STAT5A transduces extracellular signals to the nucleus and directly regulates the expression of specific target genes. This transcriptional activation is strictly dependent on phosphorylation at two critical residues: Tyr694 and Ser726. Phosphorylation of Tyr694 enables STAT5A to translocate into the nucleus and bind DNA [35]. Phosphorylation at Ser726 further amplifies the transcriptional activity of STAT5A, ensuring robust expression of its target genes [36]. In the absence of Ser726 phosphorylation, STAT5A’s ability to drive gene transcription is significantly compromised, even when Tyr694 is phosphorylated. BCLAF1 (Bcl-2-associated transcription factor 1) is a multifunctional nuclear protein that was originally identified through its interaction with the adenoviral Bcl-2 homolog E1B19K [37]. Subsequent studies have revealed the extensive roles of BCLAF1 in various processes, ranging from the DNA damage response (DDR), pre-mRNA splicing, and T cell activation to lung development, muscle cell proliferation/differentiation, autophagy, ischemia–reperfusion injury, and viral infection [38]. Moreover, emerging evidence identifies Bclaf1 as a host factor involved in anti-herpesviral defense, which is strikingly targeted by multiple viral components. The betaherpesvirus human cytomegalovirus (HCMV) employs multiple viral components—including the proteins pp71 and UL35, as well as a microRNA—to reduce cellular Bclaf1 levels [39]. However, whether Bclaf1 plays a role in alphaherpesvirus infection and the molecular mechanism of its antiviral activity remains to be elucidated.

In contrast to the pK^−^ mutant, which primarily impacted the ‘Spliceosome’ and ‘DNA replication’, the TK^−^ mutant was more strongly associated with the activation of pathways including ‘ATP-dependent chromatin remodeling’, ‘Wnt signaling’, and ‘Neurotrophin signaling’. This indicates that distinct viral mutations influence the host through different molecular mechanisms. Furthermore, a group of molecules—including SMARCA5 and RSF1—which are linked to the ATP-dependent chromatin remodeling pathway and exhibit altered phosphorylation, merits future study.

SMARCA5 serves as the catalytic ATPase subunit in the ISWI family of ATP-dependent chromatin remodeling complexes. It utilizes energy from ATP hydrolysis to mediate short-range nucleosome sliding along DNA [40]. During infection by large DNA viruses such as HCMV and adenovirus, SMARCA5 is recruited to viral replication centers, where it facilitates viral genome replication—likely by maintaining chromatin structure around replication forks through its nucleosome remodeling activities [41,42]. During infection by viruses like HSV-1 or IAV, nuclear phosphorylated RPSA recruits SMARCA5, a process that enhances chromatin accessibility at NF-κB target gene promoters and facilitates the transcription of pro-inflammatory cytokines [43]. The RSF complex, an ISWI-family chromatin remodeler, uses energy derived from ATP hydrolysis—catalyzed by its SNF2H subunit—to slide nucleosomes along DNA [44]. This repositioning alters nucleosome arrangement while preserving their structural integrity. The RSF complex possesses ATP-dependent chromatin remodeling activity. It slides nucleosomes to expose or mask DNA regions, thereby regulating transcription factor binding and gene expression [45]. During HPV infection, the RSF complex (comprising RSF1 and SNF2H) is recruited by the viral E2 protein to participate in viral chromatin regulation, thereby potentially influencing the balance between viral gene expression and silencing [46]. Therefore, further investigation into the relationship between FHV-1 replication and the RSF complex is warranted to advance our understanding of viral replication mechanisms.

Although our study focused on F81 cells infected with various FHV-1 mutants, the vast majority of identified proteins exhibit highly conserved sequences and functions across species. Thus, these findings may be broadly relevant to phosphorylation studies in other mammalian cells infected with related viruses.

## Figures and Tables

**Table 1:** Sequences of the sgRNAs.

Gene	Primers	Sequence (5′-3′)
pK	sgRNA1-pK-F	caccgcgtggtatgctggacgatga
	sgRNA1-pK-R	aaactcatcgtccagcataccacg
	sgRNA2-pK-F	caccgcactctaaaggttcacccag
	sgRNA2-pK-R	aaacctgggtgaacctttagagtg
TK	sgRNA1-TK-F	caccgcatttacatagatggtgcct
	sgRNA1-TK-R	aaacaggcaccatctatgtaaatg
	sgRNA2-TK-F	caccgccttgggagcactgagacaa
	sgRNA2-TK-F	aaacttgtctcagtgctcccaagg

**Table 2:** Primers used to generate mutating fragments.

Primer	Sequence (5′–3′)	Used for
pKL-F	gaccatgattacgccaagcttgtctcgttgtcactctcgct	generation of pK^−^ mutating fragment
pKL-R	ttaccgtaagttatgtaacgggatccagtagagatcatcg	
pK-EGFP-F	cgttacataacttacggtaa	
pK-EGFP-R	gaacttggcatcgaaagtcataagatacattgatgagttt	
pKR-F	tgactttcgatgccaagttc	
pKR-R	aaacgacggccagtgaattcgttatgtatatcctcctccg	
TKL-F	accatgattacgccaagcttggttaacggacgatctgtga	generation of TK^−^ mutating fragment
TKL-R	ttaccgtaagttatgtaacgcgtctgatctgtgtatgatg	
TK-EGFP-F	cgttacataacttacggtaa	
TK-EGFP-F	atagggaacaccactaatgttaagatacattgatgagttt	
TKR-F	acattagtggtgttccctat	
TKR-R	aaacgacggccagtgaattcgcattccatcggccagtaat	

**Table 3:** Primers used to differentiate between wild-type and mutant viruses.

Primers	Sequence (5′–3′)	Used for
pK-F1	ccacacccatatcaacatc	Differentiation of pK^−^ mutant and wild type
pK-R	gtaacagagatccattgg	
TK-F1	aacctcacatgctaggtaca	Differentiation of TK^−^ mutant and wild type
TK-R1	ccagtcaacatcctcgatac	

**Table 4:** Mass Spectrometry Gradient.

Time (min)	Flow (μL/min)	%B
0	0.4	6.7
0.7	0.4	6.7
1	0.4	7.2
11.3	0.4	24
17	0.4	36
18.5	0.4	55
19	0.4	99
24	0.4	99

**Table 5:** Mass Spectrometry Parameter Settings Table.

Properties of Full MS	
General	
Runtime (min)	24
Polarity	Positive
Full MS	
Resolution	240,000
AGC target (%)	500
Maximum Injection Time (ms)	5
Scan Range (m/z)	380–980
Properties of DIA	
General	
Runtime (min)	24
Polarity	Positive
Default charge state	2
Advanced Peak Determination	Ture
MS2	
Resolution	80,000
Precursor Mass Range (*m*/*z*)	380–980
DIA Window Type	Auto
Isolation Window (*m*/*z*)	2
Number Of Scan Events	299
DIA Window Mode	*m*/*z* Range
Collision Energy Type	Normalized
HCD Collison Energy (%)	25
Detector Type	Astral
Scan Range (*m*/*z*)	150–2000
AGC target (%)	500
Maximum Injection Time (ms)	3
Microscans	1
Loop Control	Time
Time (s)	0.6

## Data Availability

The original contributions presented in this study are included in the article/Supplementary Material. Further inquiries can be directed to the corresponding author(s).

## Supplementary Materials

The following supporting information can be downloaded at: https://www.mdpi.com/article/10.3390/vetsci12121185/s1, Figures S1 and S2: Original PCR/Gel Electrophoresis pictures.
