# Supplementary figures and images for: Phosphoproteomic Analysis Reveals Differences in Intercellular Spread Among Feline Herpesvirus Type 1 Mutants
Source: Vet Sci. 2025 Dec 11;12(12):1185. doi: 10.3390/vetsci12121185 (PMC12737363; doi:10.3390/vetsci12121185)

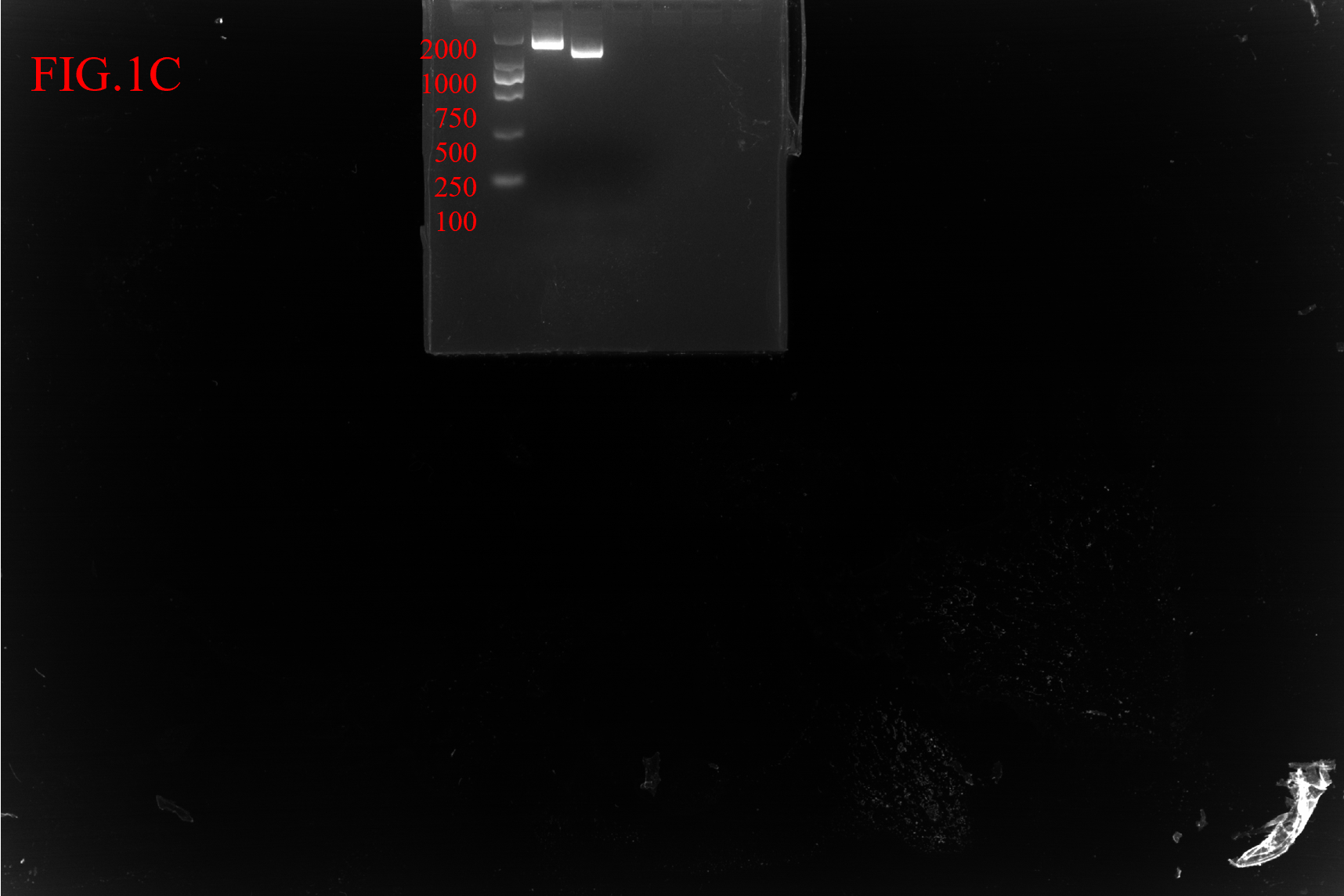

Supplement: Supplementary file 1 [file vetsci-12-01185-s001.zip › vetsci-3987926-original figures-1211/Figure S1.png]

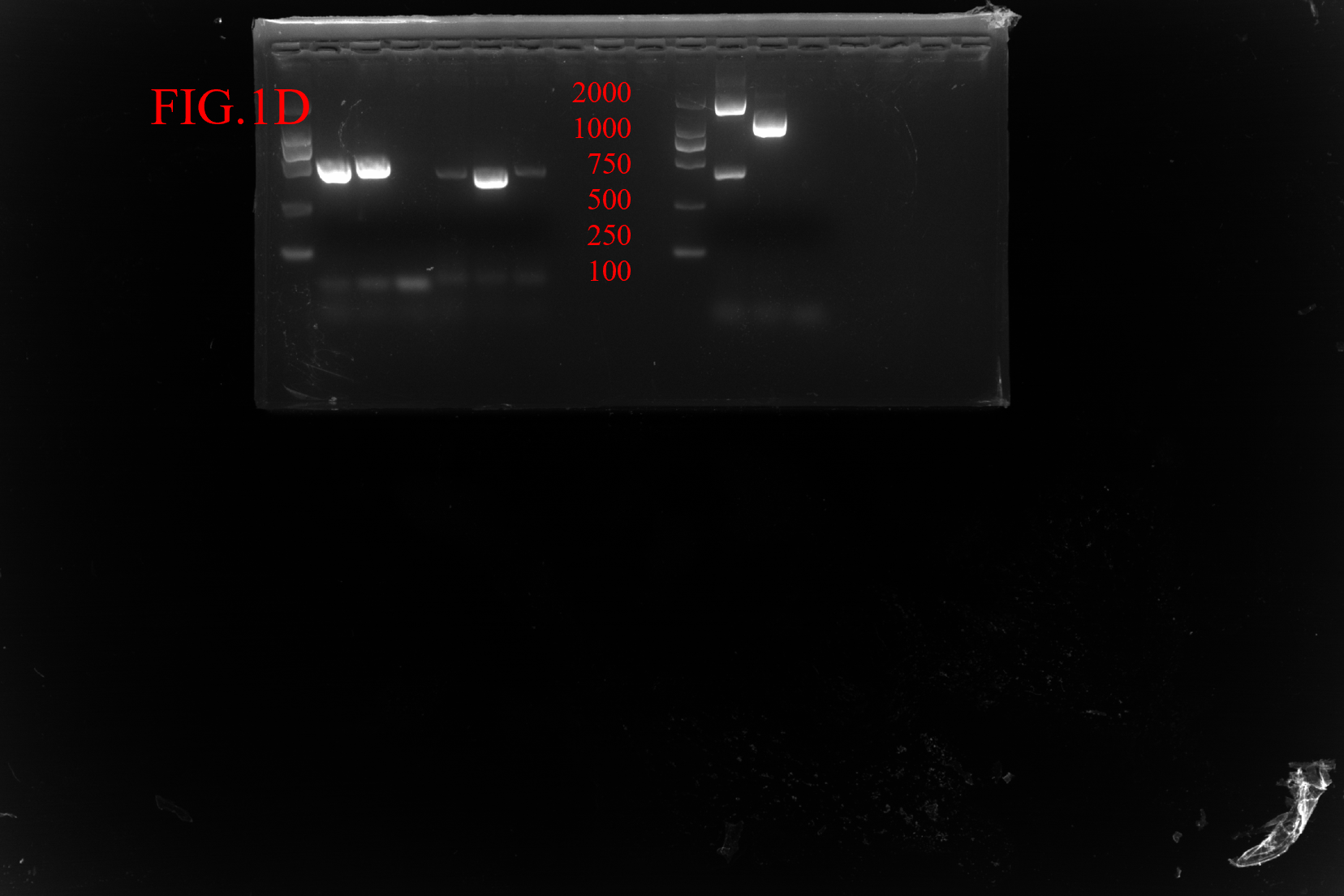

Supplement: Supplementary file 1 [file vetsci-12-01185-s001.zip › vetsci-3987926-original figures-1211/Figure S2.png]
